# Short-term anoxia improves postharvest quality and reduces browning-related enzyme activity in edible *Astraeus odoratus* mushroom

**DOI:** 10.1016/j.fochx.2026.104519

**Published:** 2026-09-25

**Authors:** Chairat Techavuthiporn, Amnat Jarerat, Helen Julian, Hataitip Nimitkeatkai

**Affiliations:** a School of Industrial Education and Technology, King Mongkut's Institute of Technology Ladkrabang, Ladkrabang, Bangkok 10520, Thailand; b Food Technology Program, Mahidol University, Kanchanaburi Campus, Saiyok, Kanchanaburi 71150, Thailand; c Faculty of Industrial Technology, Institut Teknologi Bandung, Jalan Ganesha 10, Bandung 40132, Indonesia; d School of Agriculture and Natural Resources, University of Phayao, Muang, Phayao 56000, Thailand

**Keywords:** *Astraeus odoratus*, Anoxic condition, Antioxidant capacity, Browning, Oxidative damage

## Abstract

*Astraeus odoratus*, a nutritionally and economically valuable wild edible mushroom, is highly perishable because of its high respiration rate and fragile tissue structure. This study evaluated the effects of short-term anoxic treatment prior to low-temperature storage on the postharvest quality of *A. odoratus*. Fresh mushrooms were exposed to anoxic conditions for 9 h and stored at 10 ± 2 °C. Anoxia significantly reduced weight loss, electrical conductivity, and hydrogen peroxide accumulation compared with the control. Anoxia-treated samples exhibited higher antioxidant capacity as determined by DPPH and FRAP assays. Furthermore, short-term anoxia suppressed the activities of polyphenol oxidase and peroxidase, the major enzymes associated with enzymatic browning. These results demonstrate that short-term anoxic treatment can enhance antioxidant defense, delay browning, and preserve the quality of *A. odoratus* mushrooms, highlighting the novelty of this approach for extending shelf life and promoting their commercial utilization.

## Highlights

• Eco-friendly short-term anoxia improved postharvest quality of *A. odoratus*.
• Anoxia reduced weight loss and preserved membrane integrity.
• Anoxia inhibited PPO and POD activities, delaying browning
• Anoxia treated mushrooms maintained higher levels of antioxidant activity
• Multivariate analysis confirmed the effects of anoxia on mushroom quality.

## 1 Introduction

*Astraeus odoratus*, commonly known as the barometer earthstar, is a wild edible fungus widely consumed in northern and northeastern Thailand due to its distinctive texture and flavor (Kakumyan & Matsui, 2009). It commands a higher market price than cultivated mushrooms, as its availability is limited to the wet season (Mortimer et al., 2012). *Astraeus* species exhibit nutritional compositions comparable to those of other edible mushrooms (Sanmee et al., 2003). The nutritive value of young *A. odoratus* including protein, fat, crude fiber and carbohydrate concentration were 14.0, 2.7, 10.8, and 44.9% dry weight, respectively (Sanmee et al., 2003). The crude extracts of *A. odoratus* are reported to contain bioactive compounds, antioxidant, antibacterial, antidiabetic, and anticancer properties (Ung & Chen, 2024). Among *Astraeus* species found in Thailand, *A. odoratus* has been reported to possess notably high total phenolic content and antioxidant activity (Kettawan et al., 2011).

Despite its nutritional and economic value, the postharvest quality of *A. odoratus* remains a major limitation. As an ectomycorrhizal fungus, fruiting body development depends strongly on environmental conditions, particularly rainfall and soil moisture (Pavithra et al., 2015; Phosri et al., 2004). Young fruiting bodies are off-white to grey and typically spherical in shape, whereas mature specimens become brown to dark brown, with a firmer and more compact internal structure (Kettawan et al., 2011). Exposure to sunlight and wind causes the spore-bearing outer layer hardens, resulting in a tough texture. Like many mushrooms, *A. odoratus* has a very short shelf life of approximately 1–2 days under ambient conditions, primarily due to its high respiration rate, high moisture content, and lack of protective structures to minimize water loss and microbial invasion (He et al., 2022). These factors contribute to postharvest quality deterioration, including browning, dehydration, and disruption of cellular structure (Liu et al., 2024). Although *A. odoratus* is in high demand in domestic markets, its distribution to distant markets remains limited due to its poor postharvest stability.

Mushroom senescence is primarily an oxidative process involving the degradation of cellular and subcellular structures (Wang et al., 2011). Reactive oxygen species (ROS), such as hydrogen peroxide, play a critical role in lipid peroxidation and membrane damage, thereby accelerating senescence. The susceptibility of mushroom tissues to oxidative stress depends on the balance between the generation of reactive species and the antioxidant defense capacity of the cells.

Short-term anoxic treatment has emerged as an important modified atmosphere preservation strategy. This technique involves exposing fresh produce to very low oxygen or anaerobic conditions for a limited duration, followed by storage under aerobic conditions. It has been widely reported to maintain quality and extend the shelf life of various horticultural commodities, including asparagus (Techavuthiporn & Boonyaritthongchai, 2016), pineapple (Techavuthiporn et al., 2017), broccoli (Techavuthiporn et al., 2021), litchi (Techavuthiporn et al., 2023), and banana (Sittiprasert et al., 2025; Techavuthiporn, Sittiprasert, et al., 2026; Techavuthiporn, Thammawong, & Nakano, 2026). For example, Techavuthiporn et al. (2021) demonstrated that ethanol, an anaerobic metabolite, effectively delayed yellowing in broccoli following 20 h of anoxic treatment. Similarly, Techavuthiporn et al. (2023) reported that 18 h of anoxia significantly reduced browning in litchi pericarp by suppressing polyphenol oxidase (PPO) activity, recommending this approach as a cost-effective and non-chemical pre-treatment prior to distribution. More recently, Liu et al. (2024) demonstrated that a 12 h short-term anaerobic treatment improved the postharvest quality of *Stropharia rugosoannulata*.

Despite these promising findings, excessive accumulation of anaerobic metabolites may be detrimental to tissue integrity and can lead to the development of off-odors and undesirable flavors. Therefore, optimizing anoxic treatment conditions is essential and must be tailored to specific commodities. To date, there is limited published evidence regarding the effects of postharvest treatments on wild edible mushrooms. Therefore, the present study aimed to investigate the impact of short-term anoxic treatment on the postharvest quality of *A. odoratus*. In addition, the progression of browning and the oxidative stability of mushroom tissues under anoxic conditions were systematically evaluated.

## 2 Materials and methods

### 2.1 Plant material preparations

*Astraeus odoratus* (Hed Phor) mushrooms were obtained from a local market in Phayao Province, Thailand. Fruiting bodies at the immature stage were selected for this study based on their morphological characteristics. The selected mushrooms had diameters ranging from 2.0 to 2.5 cm and an average fresh weight of 7 ± 2 g. They were characterized by a subglobose to spherical shape and firm, white, compact internal tissue*.* The mushrooms were transported to the laboratory within 2 h of purchase in a temperature-controlled vehicle. Upon arrival, the samples were visually inspected to ensure uniformity in size and color and to verify the absence of physical damage and visible microbial contamination prior to experimental use. The mushrooms were then washed thoroughly with tap water at ambient temperature to remove adhering soil and debris, followed by air-drying at ambient temperature to eliminate excess surface moisture before treatment.

### 2.2 Anoxia treatment

Preliminary experiments demonstrated that exposing mushrooms to pure nitrogen for 9 h was the most effective treatment for reducing weight loss and browning during storage. Accordingly, the mushrooms were randomly divided into two groups. Each group (1200 g) was placed in a 5 L plastic container and continuously flushed with either humidified pure N₂ (99.99%) or ambient air at a flow rate of 100 mL min^−1^ for 9 h, representing the anoxic treatment and control, respectively. The flow was maintained until the O_2_ concentration inside the container was less than 0.05% (*v*/v), as measured using a 6600-headspace gas analyzer (Illinois Instruments Inc. Illinois, IL, USA). After treatment, the mushrooms were arranged in plastic trays underlying with tissue paper and stored under controlled conditions at 10 ± 2 °C and 80–85% relative humidity for 9 days. Each treatment group consisted of twelve replicates. During the 9-day storage period, three replicates were randomly collected at 3-day intervals for the evaluation of physical attributes. For chemical and biochemical analyses, mushroom samples obtained at each sampling point were sliced, lyophilized using a lyophilizer (ScanVac CoolSafe Freeze Dryer, LaboGene, Denmark) following the protocol described by Tarafdar et al. (2017), and stored at −18 °C until further analysis.

### 2.3 Color, weight loss and browning pigment measurements

The outer and inner surface color of the mushrooms was determined using a colorimeter (CR-100 Chroma Meter, Konica Minolta Sensing Inc., Tokyo, Japan) calibrated against a standard white plate. The lightness (L*) value was measured and recorded according to the CIE Lab* color scale.

During storage, weight loss was periodically assessed using five randomly selected mushrooms from each treatment. Weight loss was calculated and expressed as a percentage of the initial fresh weight.

Browning pigment content was quantified using a modified procedure described by Techavuthiporn et al. (2023). Briefly, 0.5 g of mushroom tissue was homogenized in 80% methanol, followed by centrifugation at 10,000 ×*g* for 5 min. The absorbance of the supernatant was measured at 420 nm using a UV–Vis spectrophotometer (Shimadzu, Japan), and the results were used as an index of browning intensity.

### 2.4 Respiration rate measurements

The respiration rate of the mushrooms was determined by enclosing pre-weighed samples in a 1.5-L airtight plastic chamber. Following a 1-h incubation period to allow the accumulation of CO₂ in the tissue diffuse into the air, headspace O₂ and CO₂ concentrations were analyzed using a gas analyzer (Model 6600 Infrared Headspace Analyzer, Illinois Instruments, Systech Instruments Ltd., U.K.). The rates of O₂ consumption and CO₂ production were expressed as mL O₂·kg^−1^·h^−1^ and mL CO₂·kg^−1^·h^−1^, respectively.

### 2.5 Electrolyte leakage, malondialdehyde (MDA), and hydrogen peroxide (H_2_O_2_) determination

Electrolyte leakage was assessed by immersing 2 g of sliced mushroom tissue in deionized water at ambient temperature for 30 min. The initial electrical conductivity was measured using an EC tester (LAQUAtwin, Horiba Ltd., Kyoto, Japan). The samples were subsequently heated at 100 °C for 10 min and cooled to room temperature prior to measuring the final electrical conductivity. Electrolyte leakage was expressed as the percentage of initial conductivity relative to final conductivity.

The MDA content was determined using a modified method based on Sittiprasert et al. (2025). Briefly, 1 g of lyophilized mushroom sample was homogenized in 10% (*w*/*v*) trichloroacetic acid (TCA) and centrifuged, after which the supernatant was collected. The extract was then heated at 100 °C for 20 min and rapidly cooled prior to measuring the absorbance at 532 and 600 nm using a UV–Vis spectrophotometer. MDA content was subsequently calculated using the following equation:

$$\mathrm{MDA}=\frac{\left(\mathrm{OD}532-\mathrm{OD}600\;\right)\times V}{155\times W}$$

where V is the total volume of extract (mL), W is the sample weight (g), and 155 mM^−1^ cm^−1^ is the extinction coefficient.

Hydrogen peroxide (H₂O₂) content was determined by a modified spectrophotometric assay. Lyophilized mushroom tissue (500 mg) was homogenized on ice in 0.1% (*w*/*v*) trichloroacetic acid and centrifuged at 10,000 ×*g* for 10 min. The supernatant was subsequently combined with 0.5 mL of 10 mM potassium phosphate buffer (pH 7.0) and 1 mL of 1 M potassium iodide and incubated at room temperature for 20 min. The absorbance of the reaction mixture was measured at 390 nm using a UV–Vis spectrophotometer, and the H₂O₂ concentration was determined from a calibration curve generated using standard H₂O₂ solutions.

### 2.6 Determination of total phenolic content, DPPH scavenging and FRAP

Total phenolic content was quantified using a modified Folin–Ciocalteu assay as described by Jarerat et al. (2022). Briefly, 500 mg of lyophilized mushroom tissue was extracted with 80% methanol and centrifuged at 10,000 ×*g* for 10 min. The resulting supernatant was reacted with Folin–Ciocalteu phenol reagent, followed by the addition of 1 mL of saturated Na₂CO₃ solution. After incubation at room temperature for 90 min, the absorbance was recorded at 760 nm using a UV–Vis spectrophotometer. The results were expressed as milligrams of gallic acid equivalents per 100 g of dry weight (mg GAE·100 g^−1^ DW).

Antioxidant activity was determined using the DPPH (2,2-diphenyl-1-picrylhydrazyl) radical scavenging assay following the method of Brand-Williams et al. (1995), with minor modifications. Mushroom extracts were prepared according to the procedure described for total phenolic content determination. An aliquot of the extract was reacted with DPPH solution prepared in methanol, and the absorbance was recorded at 515 nm against a reagent blank using a UV–Vis spectrophotometer. DPPH radical scavenging activity was expressed as a percentage of inhibition.

Ferric reducing antioxidant power (FRAP) was evaluated according to a modified procedure described by Jarerat et al. (2022). The FRAP reagent consisted of 300 mM acetate buffer (pH 3.6), 10 mM TPTZ (2,4,6-tripyridyl-*s*-triazine), and 20 mM FeCl₃·6H₂O. The mushroom extract was mixed with the freshly prepared FRAP reagent, and the absorbance was recorded at 593 nm using a UV–Vis spectrophotometer. Quantification was performed using a calibration curve prepared from FeSO₄ standard solutions, and the results were expressed as μmol Fe (II)·g^−1^ DW.

### 2.7 Determination of polyphenol oxidase (PPO) and peroxidase (POD) activities

PPO and POD were extracted from 500 mg of lyophilized mushroom tissue using mM phosphate buffer (pH 6.5) and assayed according to Techavuthiporn et al. (2023) with slight modifications. The samples were homogenized and centrifuged at 10,000 ×*g* for 10 min, and the resulting supernatant was collected as the crude enzyme extract. PPO activity was determined using 70 mM catechol as the substrate. Briefly, 75 μL of the enzyme extract was added to the reaction mixture, and the increase in absorbance at 420 nm was monitored for 3 min using a UV–Vis spectrophotometer. One unit of PPO activity was defined as the amount of enzyme causing an increase in absorbance of 0.001 min^−1^ at 420 nm under the assay conditions.

For the POD activity assay, the reaction mixture consisted of 100 mM guaiacol as the substrate, 10 mM H₂O₂, and 0.5 mL of crude enzyme extract. The increase in absorbance at 470 nm was monitored for 3 min using a UV–Vis spectrophotometer. One unit of POD activity was defined as the amount of enzyme causing an increase in absorbance of 0.001 min^−1^ at 470 nm under the assay conditions. Both PPO and POD activities were expressed as Unit·g^−1^ DW.

### 2.8 Statistical analysis

Data are expressed as mean ± standard deviation (SD) of three independent replicates. Differences between the two groups were analyzed using an independent-samples *t*-test, with statistical significance determined at the 95% confidence level (*p* < 0.05). Statistical analyses were performed using SPSS Statistics version 18 (SPSS Inc., Chicago, IL, USA).

Pearson correlation analysis was performed to assess the strength and direction of linear relationships among the measured quality attributes. Pearson correlation was selected because the variables were continuous quantitative measurements and the analysis aimed to evaluate linear associations among the quality attributes. Principal component analysis (PCA) was subsequently performed to summarize the multivariate relationships among the variables. Correlation analysis and PCA were conducted using OriginPro 2025 (OriginLab Corporation, Northampton, MA, USA).

## 3 Results and discussion

### 3.1 Effect of anoxia on lightness, browning pigment and weight loss of mushroom

In general, color of mushrooms undergoes a gradual change from grey to dark brown during the postharvest period. Lightness of *A. odoratus* decreased over storage time in all groups both internal and external surface area, the value ranged from 27.79 to 31.24 and 74.34 to 91.60 in external and internal color, respectively (Fig. 1A and B). The lower L* value of external color of mushroom caused by the fruit body of immature *A. odoratus* is covered by a smooth whitish mycelial layer which may be partially encrusted with debris and turn into grey to tan color (Fig. 1D). Whereas, the interior of the spore case, the gleba, is white and solid when young and becomes brown as the mushroom matures (Hayat, 2014). The *L* * value of internal color was significantly higher in anoxia-treated samples on day 6 and 9 of storage (*p* < 0.05) (Fig. 1B). *L* * value of external color followed a similar trend but showed no significant difference between treatments, although slightly higher values were observed in anoxia-treated mushrooms (Fig. 1A).

**Fig. 1:**
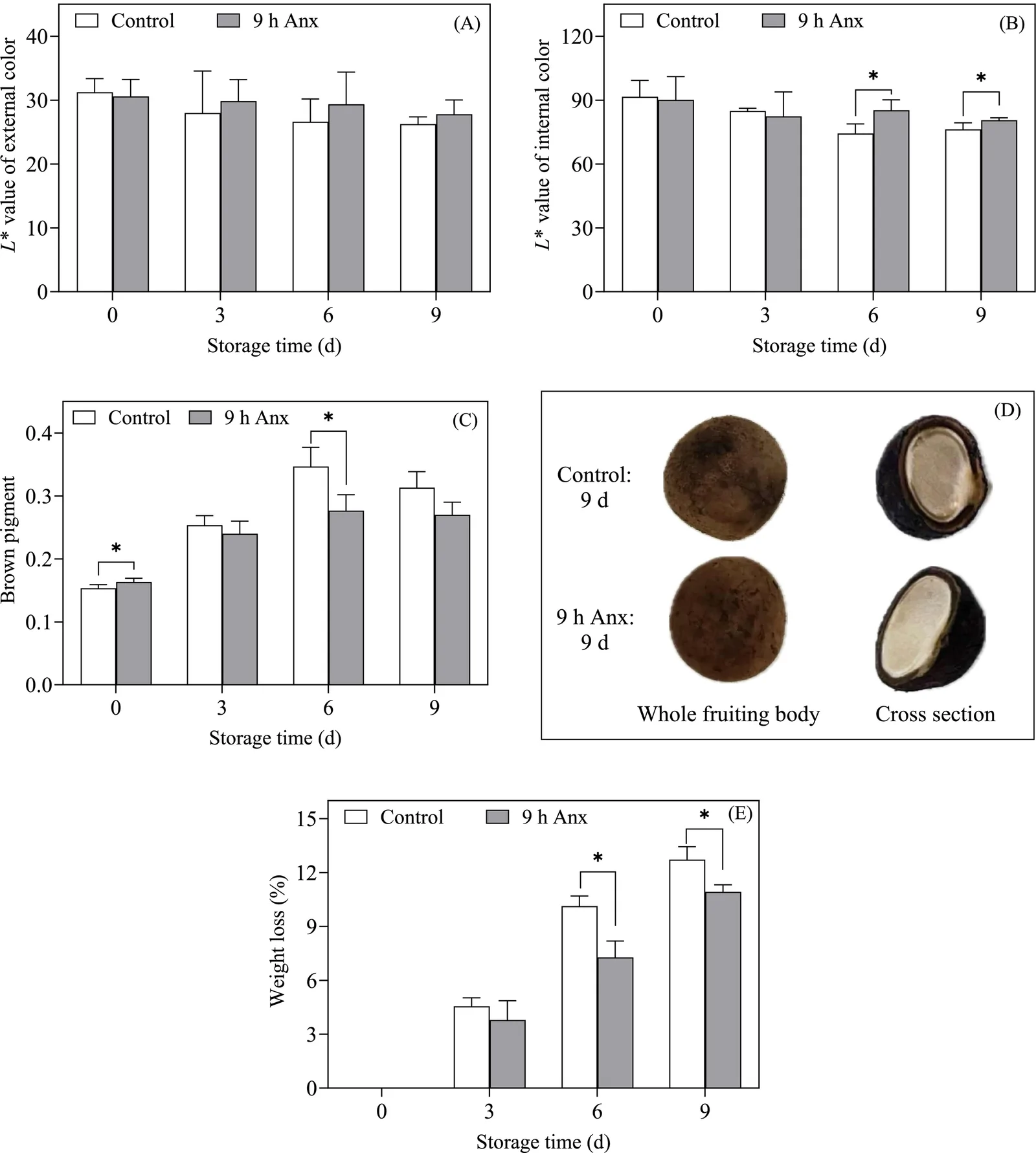
Changes in lightness of external (A) and internal (B) fruiting body, browning pigment (C), visual depiction (D), and weight loss (E) of *Astraeus odoratus* mushrooms subjected to short-term anoxia for 9 h compared with the control during storage at 10 ± 2 °C. * indicates statistically significant differences between treatments at *p* < 0.05.

The control samples exhibited the highest BI after 9 days of storage. Although the difference between control and anoxic treatment was not statistically significant, anoxia demonstrated a slightly lower browning rate (Fig. 1D), likely due to reduced oxygen availability and decreased respiration.

This browning phenomenon is primarily attributed to water loss, microbial contamination, and enzymatic activity, which predominantly occurs on the surface in the presence of oxygen (NeysariFam et al., 2025). As shown in Fig. 1A, no significant differences in the lightness of the outer surface were observed during storage. Although a definitive theoretical basis for browning pigment formation has not been fully established, higher values are generally associated with visible discoloration and reduced freshness. This may be explained by the thin and porous epidermal structure of mushrooms, which offers limited protection against rapid superficial dehydration (Jing-jun et al., 2012).

In general, weight loss increased progressively during storage. Water loss or desiccation can also lead to changes in tissue color and the development of browning, thereby reducing the visual appeal of mushrooms (Qian et al., 2021). The experimental results showed that the control group exhibited a weight loss of 12.72 ± 0.72% at the end of the storage period, whereas the 9 h anoxia-treated group showed a lower weight loss of 10.93 ± 0.39% (*p* < 0.05) (Fig. 1E) consistent with the trend in browning development (Fig. 1C and D). This reduction may be attributed to decreased water evaporation and lower oxygen consumption associated with reduced respiration. In mushrooms, which lack a protective cuticle, moisture loss is primarily driven by transpiration and can be influenced by metabolic activity (Qian et al., 2021). A similar reduction in weight loss under low-O_2_ gas conditions was reported by Park et al. (2020), who observed lower respiration rate and reduced weight loss in mushrooms stored under argon- and nitrogen-based atmospheres. Therefore, the lower weight loss observed in the anoxia-treated samples in the present study may be related to suppressing respiratory activity during storage. In addition, preservation of cellular structure under anoxic conditions may also contribute to reduced moisture loss. Previous studies have shown that anaerobic treatment can help maintain the integrity of cellular components, including plasma membranes and organelles, thereby limiting structural disintegration in mushroom tissues (Tang et al., 2021). Maintenance of cell structure may reduce water mobility and diffusion from the tissue, which could further explain the lower weight loss observed in anoxia-treated samples.

### 3.2 Effect of anoxia on O_2_ consumption and CO_2_ production rate of mushroom

Respiration-related gas exchange in mushrooms changed during storage, as reflected by variations in O_2_ consumption and CO_2_ production rates (Fig. 2A and B). O_2_ consumption generally decreased throughout storage but showed a transient increase on day 9, particularly in the anoxia-treated samples (Fig. 2A). In contrast, CO_2_ production slightly increased during the early storage period and subsequently declined thereafter (Fig. 2B). Despite this fluctuation in respiration activity, anoxia-treated mushrooms exhibited better quality parameters including lower browning development (Fig. 1C) and lower weight loss (Fig. 1E) compared with the control. Similar transient changes in respiration-related parameters during storage have also been reported in postharvest mushrooms subjected to preservation treatments or modified atmospheres (Liu et al., 2024). This suggests that the elevated respiration rate did not necessarily accelerate deterioration under these conditions (NeysariFam et al., 2025).

**Fig. 2:**
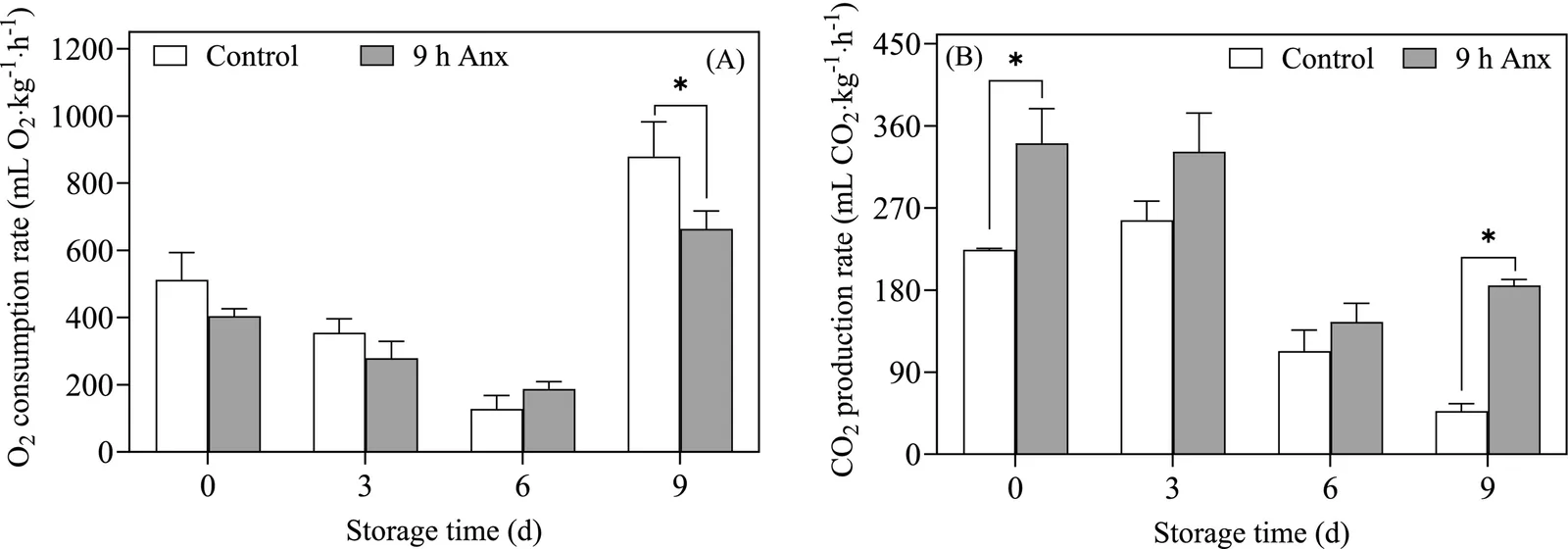
Changes in O_2_ consumption (A) and CO_2_ production rate (B) of *Astraeus odoratus* mushrooms subjected to short-term anoxia for 9 h compared with the control during storage at 10 ± 2 °C. * indicates statistically significant differences between treatments at *p* < 0.05.

Although CO_2_ production in anoxia-treated samples tended to be higher than that of the control at certain time points, the measurements of O₂ consumption and CO₂ production alone cannot distinguish aerobic respiratory responses from possible fermentative metabolism following anoxia treatment. Since the fermentative metabolites such as ethanol and acetaldehyde were not determined, no conclusion can be drawn regarding the occurrence or contribution of fermentative metabolism in *A. odoratus*. Therefore, the observed changes in O₂ consumption and CO₂ production are interpreted only as treatment-associated changes in gas-exchange behavior during subsequent aerobic storage.

### 3.3 Effect of anoxia on electrolyte leakage, malondialdehyde and hydrogen peroxide content of mushroom

Electrolyte leakage increased progressively during storage in both treatments (Fig. 3A), rising from approximately 19.19 to 59.57% in the control by the end of storage. In contrast, anoxia-treated samples showed a lower increase, reaching approximately 11.65 to 49.31% at the final stage. The difference between treatments became more evident after mid-storage, indicating delayed membrane deterioration under anoxia. A slightly higher conductivity observed in anoxia-treated samples at day 3 may reflect a transient response to oxygen deprivation.

**Fig. 3:**
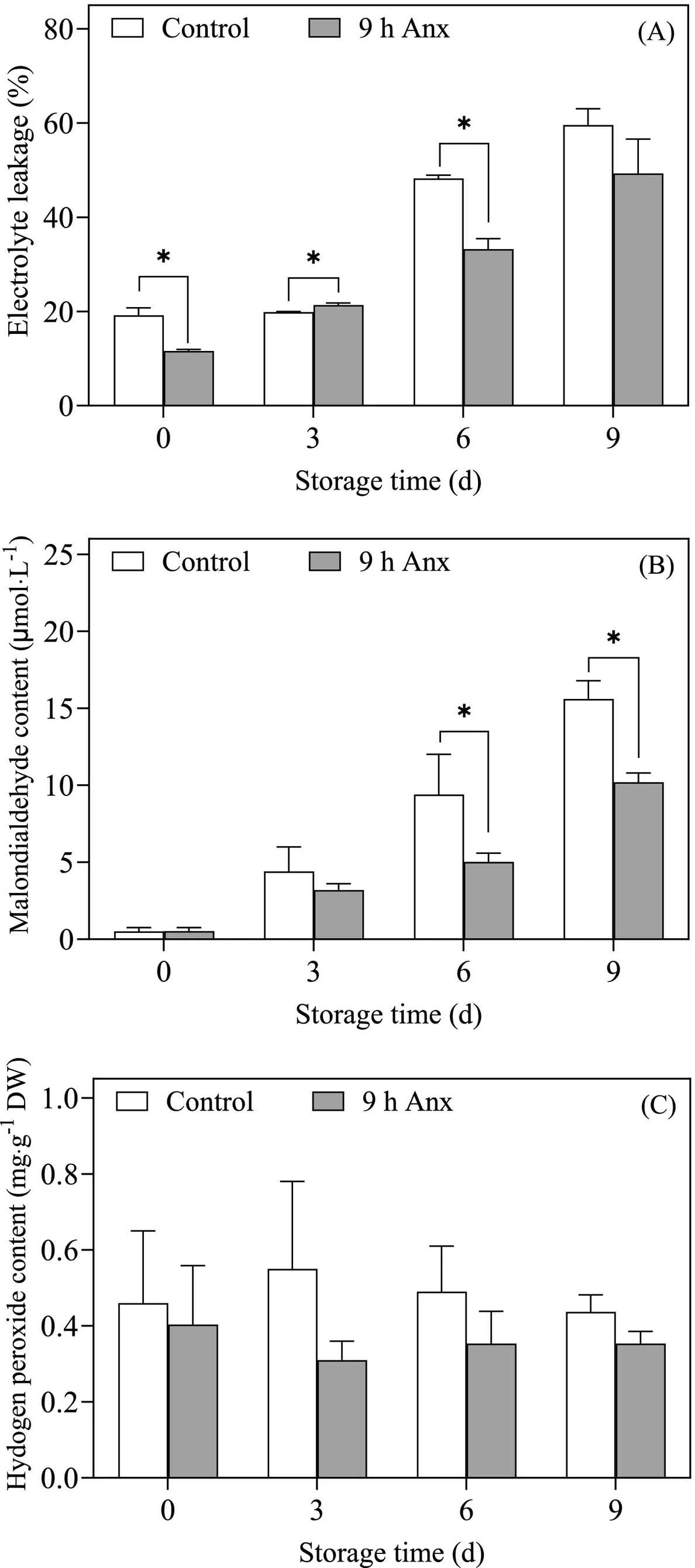
Changes in electrolyte leakage (A), malondialdehyde (B), and hydrogen peroxide (C) of *Astraeus odoratus* mushrooms subjected to short-term anoxia for 9 h compared with the control during storage at 10 ± 2 °C. * indicates statistically significant differences between treatments at *p* < 0.05.

Similarly, MDA content increased markedly during storage (Fig. 3B). In the control, MDA levels increased from approximately 0.51 to 15.60 μmol·L^−1^, whereas anoxia-treated samples reached lower levels of around 0.53 to 10.20 μmol·L^−1^. The divergence between treatments became more pronounced at later stages, suggesting reduced lipid peroxidation under anoxia conditions. Comparable trends have been reported in mushrooms subjected to short-term anaerobic treatment (Liu et al., 2024).

H_2_O_2_ content also increased over time (Fig. 3C), with the control rising from approximately 0.46 to 0.55 mg·g^−1^ DW at day 3 and declined thereafter to 0.44 mg·g^−1^ DW, while anoxia-treated samples remained lower at approximately 0.35 mg·g^−1^ DW at the final stage. Although both treatments exhibited an increasing trend, H_2_O_2_ accumulation was consistently lower under anoxia, particularly at peak levels, suggesting a moderated oxidative status. Previous studies have reported that low-O_2_ environments can enhance antioxidant defense systems and reduce oxidative stress in mushrooms (Tang et al., 2022).

Taken together, these results indicate that anoxia treatment mitigated oxidative damage and delayed membrane deterioration during storage. Browning development in mushrooms is influenced not only by phenolic compounds and browning-related enzymes, but also by membrane lipid peroxidation and loss of membrane integrity (Liang et al., 2024). Previous studies have shown that increases in MDA content and relative conductivity are closely linked to browning progression in postharvest mushrooms (Liu et al., 2024). Therefore, the lower electrolyte leakage and MDA accumulation observed in anoxia-treated samples may contribute to the reduced browning (Fig. 1B and C) and lower weight loss (Fig. 1E), as preservation of membrane integrity can limit enzymatic browning and moisture loss. The regulation of oxidative status under anoxia conditions may involve changes in antioxidant systems and browning-related enzymes, which are further discussed in the following section.

### 3.4 Effect of anoxia on PPO and POD activities of mushroom

Changes in polyphenol oxidase (PPO) and peroxidase (POD) activities during storage are shown in Fig. 4. PPO activity increased progressively in both treatments during storage at 10 °C (Fig. 4A). However, the increase was more pronounced in the control, whereas mushrooms subjected to 9 h anoxia exhibited consistently lower PPO activity throughout storage (*p* < 0.05). The difference between treatments became more evident at the later stages, where the control reached the highest PPO activity. PPO is considered a key enzyme responsible for enzymatic browning in mushrooms (NeysariFam et al., 2025). This role of PPO is well established across various plant tissues, where its activity is closely associated with discoloration development (Techavuthiporn & Supapvanich, 2024). In addition, anoxia treatment evidently inhibited the increasing PPO activity during storage in postharvest tissues (Supapvanich & Techavuthiporn, 2022; Techavuthiporn et al., 2017). This inhibitory effect is likely attributed to limited oxygen availability during and after short-term anoxia treatment, as PPO activity is strongly dependent on molecular oxygen (Tang et al., 2022). Moreover, reduced PPO activity has been associated with decreased melanin formation, thereby effectively suppressing browning development in mushrooms (Fu et al., 2022).

**Fig. 4:**
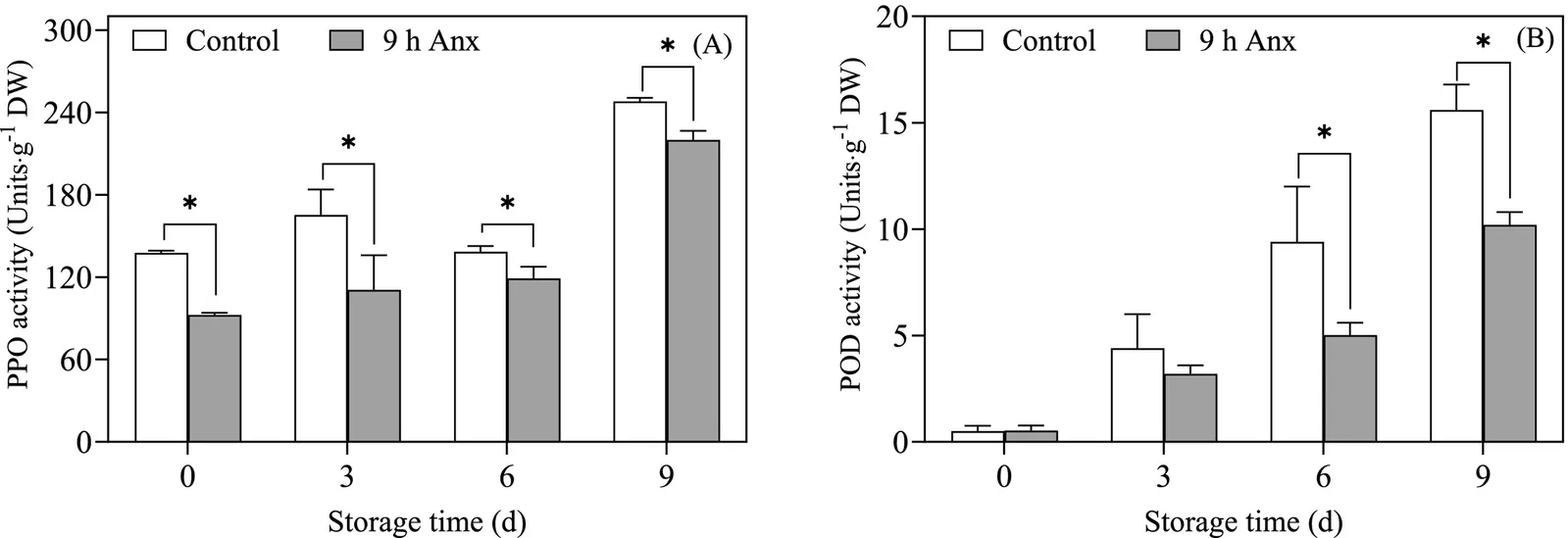
Changes in PPO (A) and POD (B) activities of *Astraeus odoratus* mushrooms subjected to short-term anoxia for 9 h compared with the control during storage at 10 ± 2 °C. * indicates statistically significant differences between treatments at *p* < 0.05.

NeysariFam et al. (2025) suggested that POD activity is closely associated with senescence and oxidative processes in mushrooms. POD is one of the main enzymes that oxidize phenolic substrates into anthraquinone compounds that cause the occurrence of browning (Qian et al., 2021). In the present study, a similar trend was observed for POD activity (Fig. 4B). POD activity increased markedly during storage in both treatments, but the control samples showed a higher rate of increase compared with anoxia-treated mushrooms (*p* < 0.05). Although POD activity in anoxia-treated samples also increased over time, it remained consistently lower than that of the control, particularly at the final stage of storage. The lower POD activity observed in anoxia-treated mushrooms may be associated with altered oxidative status under low-O_2_ conditions, as reduced oxygen availability can influence oxidative metabolism in postharvest tissues (Min et al., 2019). Given that POD is involved in H_2_O_2_-dependent oxidative reactions, the reduced H_2_O_2_ accumulation observed in this study may have limited POD activity during storage. This suggests that modulation of reactive oxygen species under anoxia could play a role in regulating browning-related enzyme activity.

Overall, the lower PPO and POD activities observed in anoxia-treated mushrooms suggest a suppression of browning-related enzyme activity after anoxia treatment. This trend is consistent with the reduced browning development observed in Fig. 1, indicating that anoxia treatment may contribute to maintaining visual quality during storage. This behavior is also in line with the reduced oxidative damage and improved membrane integrity observed in Fig. 3.

### 3.5 Effect of anoxia on total phenolic content and antioxidant capacities of mushroom

Changes in total phenolic content (TPC), DPPH radical scavenging activity, and ferric reducing antioxidant power (FRAP) during storage at 10 °C are shown in Fig. 5. TPC varied during storage in both the control and anoxia-treated mushrooms (Fig. 5A). On day 0, the anoxia-treated mushrooms had significantly lower TPC than the control (*p* < 0.05). However, no significant differences in TPC between the control and anoxia-treated mushrooms were observed on days 3, 6, and 9 (*p* ≥ 0.05). Although the mean TPC values tended to be lower in the anoxia-treated mushrooms on day 3 and higher on days 6 and 9, these differences were not statistically significant. Therefore, the results indicate that the 9-h anoxia treatment did not consistently maintain higher TPC during subsequent storage. Phenolic compounds are recognized as major naturally occurring antioxidants in edible mushrooms (Abdelshafy et al., 2022), and changes in TPC during storage may be associated with several biochemical processes, including oxidation of phenolic compounds. PPO-mediated oxidation can contribute to phenolic loss in the presence of oxygen (Yan et al., 2020). However, because no significant differences between treatments were observed after day 0, the present results do not provide sufficient evidence to conclude that the anoxia treatment preserved phenolic compounds during subsequent aerobic storage.

**Fig. 5:**
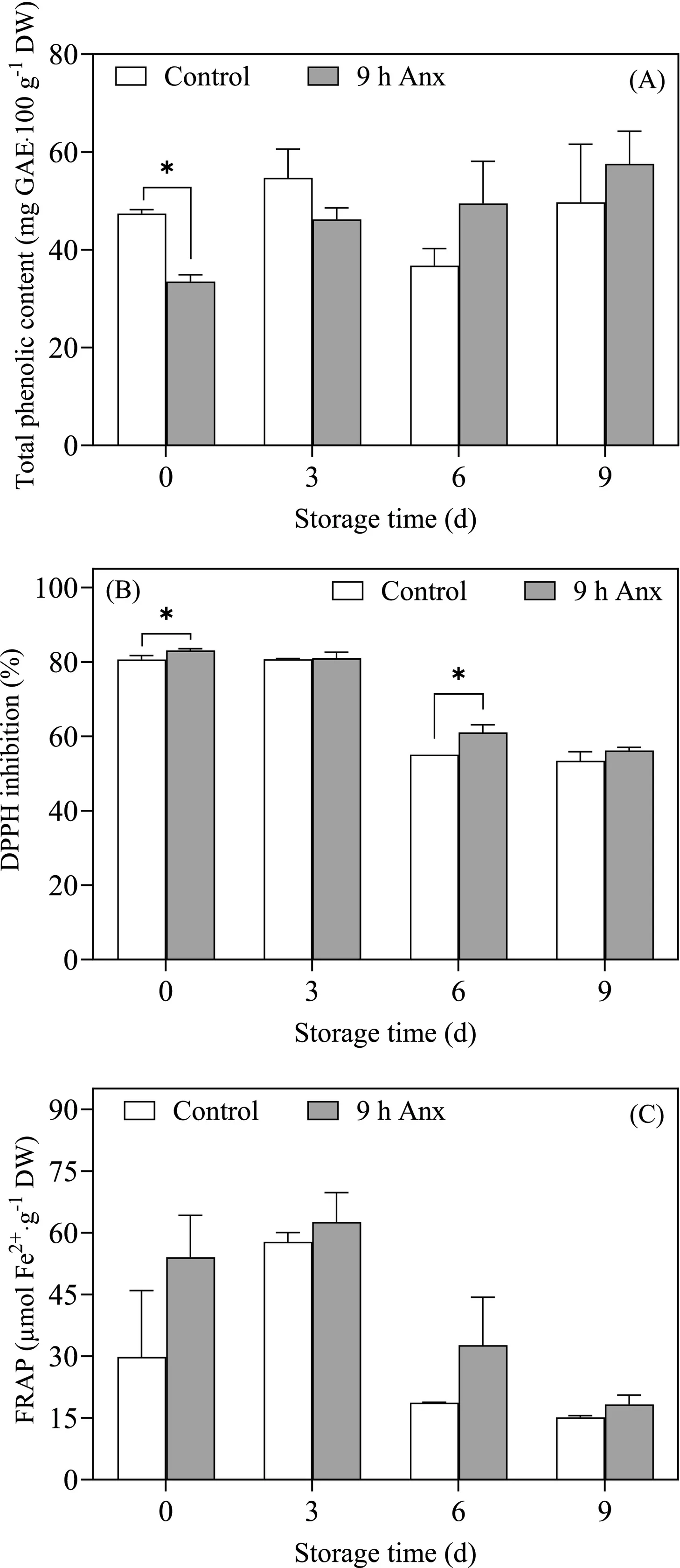
Changes in total phenolic content (A), DPPH radical scavenging activity (B) and FRAP (C) of *Astraeus odoratus* mushrooms subjected to short-term anoxia for 9 h compared with the control during storage at 10 ± 2 °C. * indicates statistically significant differences between treatments at *p* < 0.05.

Antioxidant activity was evaluated using DPPH and FRAP assays. The DPPH assay reflects the ability of antioxidants to scavenge free radicals, whereas the FRAP assay measures the reducing power of the sample by converting Fe^3+^ to Fe^2+^ (Lin et al., 2017). DPPH scavenging activity increased at the early stage and decreased thereafter in both treatments (Fig. 5B), with anoxia-treated samples consistently showing higher activity than the control. In contrast, FRAP values followed a similar trend but showed no significant difference between treatments, although slightly higher values were observed in anoxia-treated mushrooms (Fig. 5C). The enhanced antioxidant capacity observed in anoxia-treated mushrooms may be associated with modulation of oxidative metabolism under low-O_2_ conditions. Changes in respiratory metabolism under anaerobic environments have been suggested to influence oxidative status and cellular stability in postharvest mushrooms (Tang et al., 2021). In addition, anaerobic treatment has been shown to enhance antioxidant defense systems in horticultural products, supporting the role of oxygen limitation in regulating reactive oxygen species and antioxidant capacity (Ying et al., 2024).

### 3.6 Multivariate relationships among quality attributes under short-term anoxia

The interconnections among quality-related aspects of *A. odoratus* mushrooms during storage were initially assessed using Pearson correlation analysis (Fig. 6A) and subsequently synthesized using principal component analysis (PCA) (Fig. 6B). The correlation heatmap demonstrated clear clusters related to browning development, oxidative damage, membrane integrity, and metabolic activity, underscoring the intricate relationships that regulate postharvest quality alterations.

**Fig. 6:**
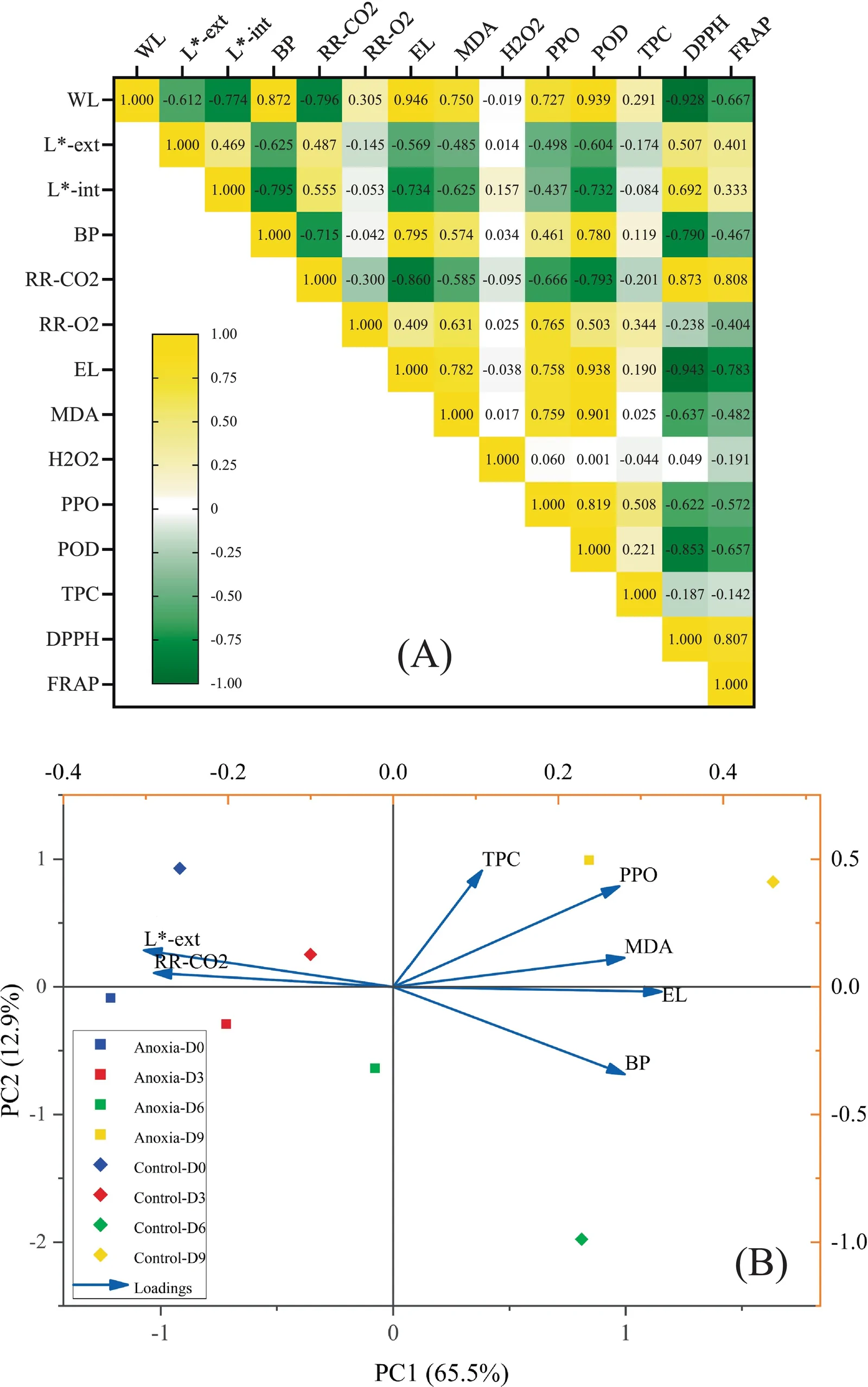
Multivariate analysis of quality attributes of *Astraeus odoratus* mushrooms subjected to short-term anoxia during storage. (A) Correlation heatmap illustrating the relationships among browning-related parameters, oxidative damage indicators, membrane integrity, metabolic activity, and antioxidant attributes. (B) Principal component analysis (PCA) biplot showing the distribution of samples and variable loadings. PC1 represents the quality deterioration and browning axis, while PC2 reflects antioxidant-related modulation. Symbols represent samples from Anoxia and Control treatments across storage days (D0–D9).

Significant positive associations were identified among polyphenol oxidase (PPO), brown pigment (BP), malondialdehyde (MDA), and electrolyte leakage (EL), suggesting that enzymatic browning, lipid peroxidation, and membrane disruption occurred simultaneously during storage (Lin et al., 2016). This trend corroborates the notion that oxidative stress-induced membrane degradation promotes enzymatic browning reactions in mushrooms, thereby hastening quality deterioration (Wang et al., 2022). Conversely, respiration rate (RR-CO_2_) had a significant negative connection with PPO, BP, and EL, indicating that reduced metabolic activity was linked to delayed browning progression and enhanced membrane stability (Li et al., 2025). Total phenolic content (TPC) exhibited modest to moderate associations with browning-related metrics, suggesting a more indirect involvement in quality preservation via antioxidant buffering rather than direct prevention of browning incidents (Serra et al., 2021; Sittiprasert et al., 2025).

The PCA biplot (Fig. 6B) coherently illustrated these correlation patterns. The first principal component (PC1, 65.5%) predominantly characterized a quality deterioration axis, with positive loadings for PPO, BP, MDA, and EL, whereas RR-CO_2_ and exterior lightness (L*-ext) were positioned on the negative side, signifying enhanced visual quality and diminished metabolic intensity. The close spatial correlation among PPO, BP, and MDA in the loading plot further substantiates the strong interconnection between oxidative damage and enzymatic browning processes. The second principal component (PC2, 12.9%) was predominantly influenced by TPC, which was aligned separately from the browning cluster, indicating that phenolic compounds principally contributed to antioxidant capacity rather than directly regulating browning severity.

The score plot exhibited a distinct differentiation between short-term anoxia-treated samples and the control, especially during the later stages of storage. Control samples were strongly linked to the browning–oxidative damage cluster, but anoxia-treated samples were situated closer to RR-CO_2_ and TPC, indicating less metabolic activity and increased oxidative stability. The integrated correlation and PCA analyses indicate that short-term anoxia significantly influenced metabolic, oxidative, and enzymatic pathways, consequently postponing browning progression and maintaining the overall quality of *A. odoratus* mushrooms during storage (Tang et al., 2021).

## 4 Conclusions

Short-term anoxia treatment for 9 h reduced browning and maintaining quality of *A. odoratus* mushroom during storage at 10 ± 2 °C. Treated mushrooms maintained higher levels of antioxidant activity and lower levels of MDA and H_2_O_2_ content, PPO and POD activities during postharvest storage. Our results suggest a significant effect of reversible oxidative damage to the cells and activation of antioxidant activity of short-term anoxic treatment. These results indicate that short-term anoxia treatment for 9 h could be used as favorable treatment to extend shelf life of *A. odoratus* mushroom and represent an environmental-friendly application to be used along with low temperatures storage in mushrooms. This research provides technical support for maintaining the quality of wild edible mushrooms and offers a new postharvest approach for enhancing their bioactivity.

## CRediT authorship contribution statement

**Chairat Techavuthiporn:** Writing – review & editing, Writing – original draft, Visualization, Supervision, Methodology, Investigation, Formal analysis, Data curation, Conceptualization. **Amnat Jarerat:** Visualization, Validation, Methodology, Data curation. **Helen Julian:** Visualization, Investigation, Formal analysis. **Hataitip Nimitkeatkai:** Writing – review & editing, Writing – original draft, Visualization, Supervision, Resources, Methodology, Investigation, Funding acquisition, Data curation, Conceptualization.

## Declaration of competing interest

The authors declare that they have no known competing financial interests or personal relationships that could have appeared to influence the work reported in this paper.

## Data Availability

Data will be made available on request.

## References

[bb0005] Abdelshafy A.M., Belwal T., Liang Z., Wang L., Li D., Luo Z., Li L. (2022). A comprehensive review on phenolic compounds from edible mushrooms: Occurrence, biological activity, application and future prospective. Critical Reviews in Food Science and Nutrition.

[bb0010] Brand-Williams W., Cuvelier M.E., Berset C. (1995). Use of free radical method to evaluate antioxidant activity. LWT- Food Science and Technology.

[bb0015] Fu Y., Yu Y., Tan H., Wang B., Peng W., Sun Q. (2022). Metabolomics reveals dopa melanin involved in the enzymatic browning of the yellow cultivars of east Asian golden needle mushroom (*Flammulina filiformis*). Food Chemistry.

[bb0020] Hayat, Y. (2014). *Astraeus hygrometricus* and its various aspests. Retrieved from 10.2139/ssrn.2459395/. Accessed May 27, 2026.

[bb0025] He X., Wang X., Liu Y., Fang H., Zheng S., Liu H., Guan W., Yan R. (2022). Biodegradable PBAT/PLA packaging maintained the quality of postharvest shiitake mushroom by modified humidity and atmosphere. Food Packaging and Shelf Life.

[bb0030] Jarerat A., Techavuthiporn C., Chanchomsuek C., Nimitkeatkai H. (2022). Enhancement of antioxidant activity and bioactive compounds in eggplants using postharvest LEDs irradiation. Horticulturae.

[bb0035] Jing-jun Y.E., Jian-rong L.I., Xiao-xiang H., Lei Z., Tian-jia J., Miao X. (2012). Effects of active modified atmosphere packaging on postharvest quality of shiitake mushrooms (*Lentinula edodes*) stored at cold storage. Journal of Integrative Agriculture.

[bb0040] Kakumyan P., Matsui K. (2009). Characterization of volatile compounds in *Astraeus* spp. Bioscience, Biotechnology, and Biochemistry.

[bb0045] Kettawan A., Chanlek K., Kongkachuichai R., Charoensiri R. (2011). Effects of cooking on antioxidant activities and polyphenol content of edible mushrooms commonly consumed in Thailand. Pakistan Journal of Nutrition.

[bb0050] Li Y., Mao X., Ma Q., Yue X., Song N., Si Z., Wang S., Zhang X., Ren X., Ding Y., Ren Y. (2025). Preharvest ABA treatment reduces browning of watercore apple fruit by regulating antioxidant and energy metabolism. Food Chemistry.

[bb0055] Liang Y., Luo K., Wang B., Huang B., Fei P., Zhang G. (2024). Inhibition of polyphenol oxidase for preventing browning in edible mushrooms: A review. Journal of Food Science.

[bb0060] Lin Q., Yuanyuan L., Zhang J., Liu W., Guan W., Wang Z. (2017). Effects of high CO_2_ in-package treatment on flavor, quality and antioxidant activity of button mushroom (*Agaricus bisporus*) during postharvest storage. Postharvest Biology and Technology.

[bb0065] Lin Y., Lin H., Lin Y., Zhang S., Chen Y., Jiang X. (2016). The roles of metabolism of membrane lipids and phenolics in hydrogen peroxide-induced pericarp browning of harvested longan fruit. Postharvest Biology and Technology.

[bb0070] Liu X., Fan T., Zhang Z., Wang Y., Zhang Y., Jiang F., Sun B. (2024). Short-term anaerobic treatment with microperforated packaging enhanced harvest quality of *Stropharia rugosoannulata* mushrooms. Food Packaging and Shelf Life.

[bb0075] Min T., Liu E.C., Xie J., Yi Y., Wang L.M., Ai Y.W., Wang H.X. (2019). Effects of vacuum packaging on enzymatic browning and ethylene response factor (ERF) gene expression of fresh-cut lotus root. HortScience.

[bb0080] Mortimer P.E., Karunarathna S.C., Li Q., Gui H., Yang X., Yang X., He Y., Ye L., Guo J., Li H., Sysouphanthong P., Zhou D., Xu J., Hyde K.D. (2012). Prized edible Asian mushrooms: Ecology, conservation and sustainability. Fungal Diversity.

[bb0085] NeysariFam V., Golmohammadi A., Tabatabaekoloor R., Tahmasebi M., Nematollahzadeh A., Razavi M.S., Nowacka M., Ghaani M. (2025). Preservation of button mushrooms using modified atmosphere and bio-nanocomposite film: A combined approach. Applied Food Research.

[bb0090] Park D.H., Park J.J., Olawuyi I.F., Lee W.Y. (2020). Quality of white mushroom (*Agaricus bisporus*) under argon-and nitrogen-based controlled atmosphere storage. Scientia Horticulturae.

[bb0095] Pavithra M., Greeshma A.A., Karun N.C., Sridhar K.R. (2015). Observations on the *Astraeus* spp. of Southwestern India. Mycosphere.

[bb0100] Phosri C., Watling R., Martín M.P., Whalley A.J.S. (2004). The genus *Astraeus* in Thailand. Mycotaxon.

[bb0105] Qian X., Hou Q., Liu J., Huang Q., Jin Z., Zhou Q., Jiang T., Zheng X. (2021). Inhibition of browning and shelf life extension of button mushroom (*Agaricus bisporus*) by ergothioneine treatment. Scientia Horticulturae.

[bb0110] Sanmee R., Dell B., Lumyong P., Izumori K., Lumyong S. (2003). Nutritive value of popular wild edible mushrooms from northern Thailand. Food Chemistry.

[bb0115] Serra S., Anthony B., Boscolo Sesillo F., Masia A., Musacchi S. (2021). Determination of post-harvest biochemical composition, enzymatic activities, and oxidative browning in 14 apple cultivars. Foods.

[bb0120] Sittiprasert W., Supapvanich S., Sinthusamran S., Nimitkeatkai H., Techavuthiporn C. (2025). Pre-storage anoxia treatment induces physicochemical characteristics of ‘Sucrier'bananas (*Musa* AA group) in response to postharvest cold storage and subsequent ambient temperature. Postharvest Biology and Technology.

[bb0125] Supapvanich S., Techavuthiporn C. (2022). Efficiency of pre-process anoxia treatment on quality of fresh-cut pineapples cv. ‘Phulae’ during storage. International Journal of Food Science and Technology.

[bb0130] Tang J., Chen X., Zhang Y., Wang J., Jiang F., Sun B. (2022). The ultra-low O_2_ environment after anaerobic treatment enhanced the antioxidant properties of *Agaricus bisporus* mushroom. Scientia Horticulturae.

[bb0135] Tang J., Ren H., Chen X., Ma F., Jiang F., Sun B. (2021). Effects of short-term N2 anaerobic treatment on respiratory metabolism and oxidation status of *Agaricus bisporus*. Postharvest Biology and Technology.

[bb0140] Tarafdar A., Shahi C., Singh A., Sirohi R. (2017). Optimization of freeze-drying process parameters for qualitative evaluation of button mushroom (*Agaricus bisporus*) using response surface methodology. Journal of Food Quality.

[bb0145] Techavuthiporn C., Boonyaritthongchai P. (2016). Effect of prestorage short-term anoxia treatment and modified atmosphere packaging on the physical and chemical changes of green asparagus. Postharvest Biology and Technology.

[bb0150] Techavuthiporn C., Boonyaritthongchai P., Supabvanich S. (2017). Physicochemical changes of ‘Phulae’ pineapple fruit treated with short-term anoxia during ambient storage. Food Chemistry.

[bb0155] Techavuthiporn C., Jarerat A., Nimitkeatkai H. (2023). Short-term anoxic treatment of litchi fruit (*Litchi chinensis* Sonn. cv. Hong Huey) delays pericarp browning and maintains physicochemical quality during storage. Horticulture Journal.

[bb0160] Techavuthiporn C., Sittiprasert W., Phakawan J., Supapvanich S. (2026). Implementing short-term anoxic conditions as a straightforward and effective postharvest technique to preserve the quality of ‘Sucrier’ bananas. Acta Horticulturae.

[bb0165] Techavuthiporn C., Supapvanich S. (2024). Efficacy of coconut liquid endosperm as natural agent inhibiting browning and maintaining quality of fresh-cut mature mangoes by comparison with cysteine. International Food Research Journal.

[bb0170] Techavuthiporn C., Thammawong M., Nakano K. (2021). Effect of short-term anoxia treatment on endogenous ethanol and postharvest responses of broccoli florets during storage at ambient temperature. Scientia Horticulturae.

[bb0175] Techavuthiporn C., Thammawong M., Nakano K. (2026). Short-term postharvest anoxia delays peel degreening and firmness loss through regulation of color- and cell wall-related genes in banana. Applied Food Research.

[bb0180] Ung A.T., Chen H. (2024). Biological properties, health benefits and semisynthetic derivatives of edible *Astraeus* mushrooms (*Diplocystidiaceae*): A comprehensive review. Chemistry & Biodiversity.

[bb0185] Wang C.T., Wang C.T., Cao Y.P., Nout M.J.R., Sun B.G., Liu L. (2011). Effect of modified atmosphere packaging (MAP) with low and superatmospheric oxygen on the quality and antioxidant enzyme system of golden needle mushroom (*Flammulina velutipes*) during postharvest storage. European Food Research and Technology.

[bb0190] Wang X., Yang Z., Cui J., Zhu S. (2022). Nitric oxide made a major contribution to the improvement of quality in button mushrooms (*Agaricus bisporus*) by the combined treatment of nitric oxide with 1-MCP. Foods.

[bb0195] Yan M., Yuan B., Xie Y., Cheng S., Huang H., Zhang W., Chen J., Cao C. (2020). Improvement of postharvest quality, enzymes activity and polyphenoloxidase structure of postharvest *Agaricus bisporus* in response to high voltage electric field. Postharvest Biology and Technology.

[bb0200] Ying L., Bian J., Zhao F., Chen X., Tang J., Jiang F., Sun B. (2024). Short-term anaerobic treatment maintained the quality of *Actinidia arguta* by activating the antioxidant defense system. Journal of the Science of Food and Agriculture.

